# Supplementary material for: Cultured meat platform developed through the structuring of edible microcarrier-derived microtissues with oleogel-based fat substitute
Source: Nat Commun. 2023 May 23;14:2942. doi: 10.1038/s41467-023-38593-4 (PMC10205709; doi:10.1038/s41467-023-38593-4)
Supplement: Supplementary file 1 — Supplementary information [file 41467_2023_38593_MOESM1_ESM.pdf]

Cultured meat platform developed through the structuring of edible microcarrier-derived microtissues with oleogel-based fat substitute

Feng-Chun Yen[1#], Jovana Glusac[1#], Shira Levi[1], Anton Zernov[1], Limor Baruch[1], Maya Davidovich-Pinhas[1], Ayelet Fishman[1]*, and Marcelle Machluf[1]**

Supplementary Information

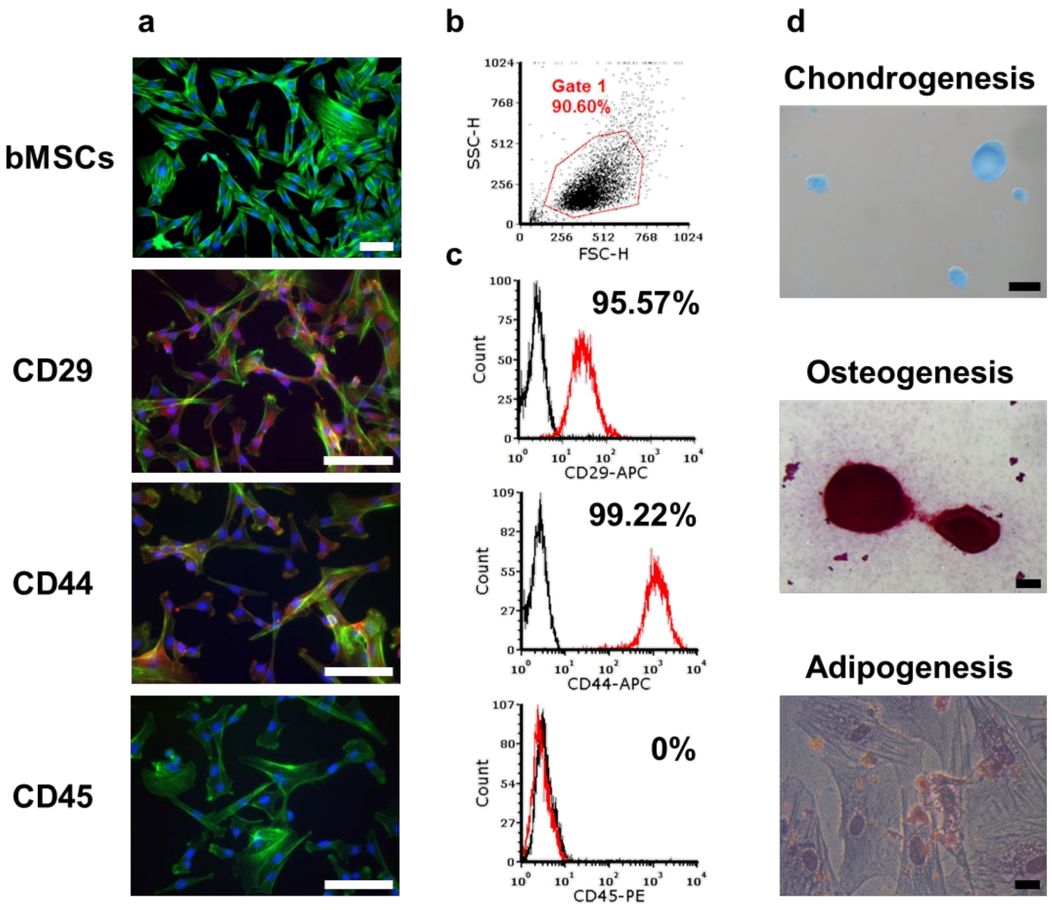

Supplementary Figure 1. Characterization of isolated bMSCs. Bovine MSCs were characterized by fluorescence staining for their morphology, and their immunophenotypic features were analyzed through immunofluorescence staining (a), representative images were from at least 3 independent samples with similar results) and flow cytometry (b-c) showing positive (CD29, CD44) and negative (CD45) bMSC surface markers. Green: Actin (Phalloidin); Red: Surface markers; Blue: Nuclei (DAPI). Scale bars: 100 µm. (b) Flow cytometry gating. (d) bMSCs differentiation potential towards chondrocytes (glycosaminoglycan accumulation, Scale bars: 250 µm), osteocytes (calcium deposits, Scale bars: 250 µm), and adipocytes (fat droplets, 20 µm). Representative images were from at least 3 independent samples with similar results. Source data are provided as a Source Data file.

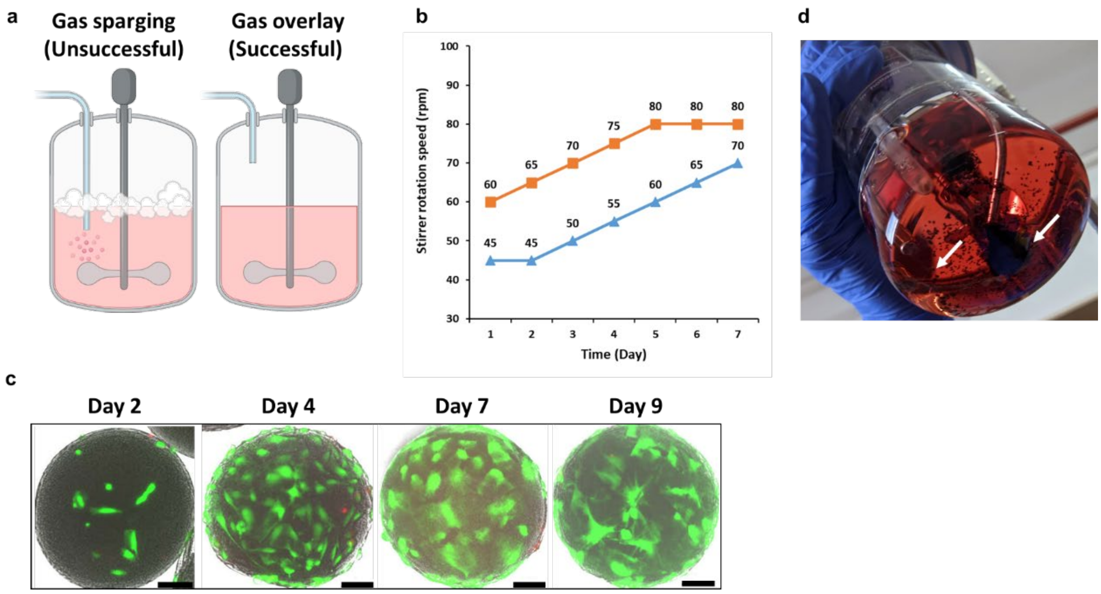

Supplementary Figure 2. Optimization of parameters for bioreactor culture. (a) Aeration methods of gas sparging or gas overlay were investigated for the bioreactor. Created with BioRender.com. (b) Two stirring regimens were applied from 45 rpm to 70 rpm or from 60 rpm to 80 rpm to the bioreactor. (c) Live/dead cell imaging of bMSC on the microcarriers expanded using a stirring regimen from 45 rpm to 70 rpm in the bioreactor. Green: live cells (FDA); Red: dead cells (PI). Representative images were from at least 5 independent samples with similar results. Scale bars: 100 µm. (d) Aggregates (arrows) formed at the bottom of the bioreactor when cultured with a stirring regimen from 45 rpm to 70 rpm.

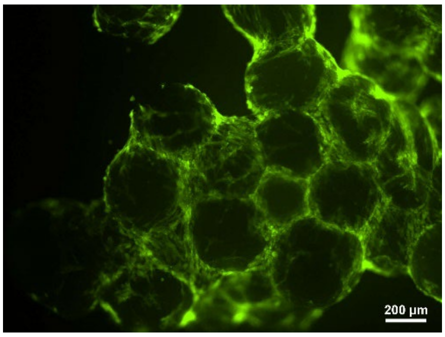

Supplementary Figure 3. Microtissue aggregation in suspension plate culture. Cellularized microtissues were aggregated during their culture in a suspension plate. Green: live cells (FDA); Red: dead cells (PI). Representative images were from at least 5 independent samples with similar results.

a

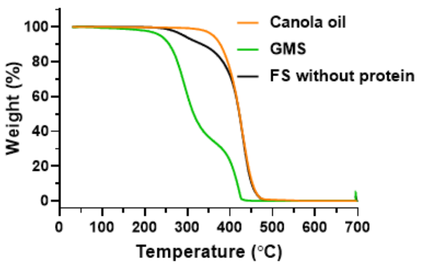

b

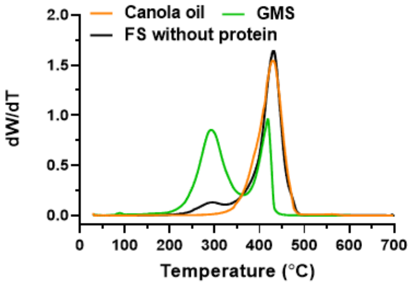

c

	FS	Beef fat
**To (°C)**	63.97 ± 1.43	35.08 ± 0.81
**Tp (°C)**	70.34 ± 0.46	42.19 ± 0.30
**ΔH (J/g)**	27.43 ± 2.49	14.51 ± 0.20

d

Samples	Hardness (N)	Adhesiveness (Nmm)	Cohesiveness	Chewiness (N)	Springiness	*L**	*a**	*b**
**Raw FS**	18.16 ± 1.03	4.25 ± 0.68	0.08 ± 0.01	1.38 ± 0.04	0.99 ± 0.01	73.34 ± 0.53	3.40 ± 0.10	20.03 ± 0.45
**Cooked FS**	31.40 ± 5.05	3.27 ± 0.93	0.04 ± 0.03	1.14 ± 0.98	0.99 ± 0.01	65.98 ± 0.16	3.86 ± 0.54	20.92 ± 0.76
**Raw beef fat**	21.52 ± 0.45	2.48 ± 1.71	0.12 ± 0.09	2.70 ± 2.12	1.00 ± 0.00	72.19 ± 1.94	6.75 ± 0.29	2.38 ± 0.04
**Cooked beef fat**	22.42 ± 3.18	1.62 ± 0.13	0.36 ± 0.01	8.05 ± 1.11	0.99 ± 0.01	49.07 ± 2.30	3.50 ± 0.84	7.01 ± 1.35

e

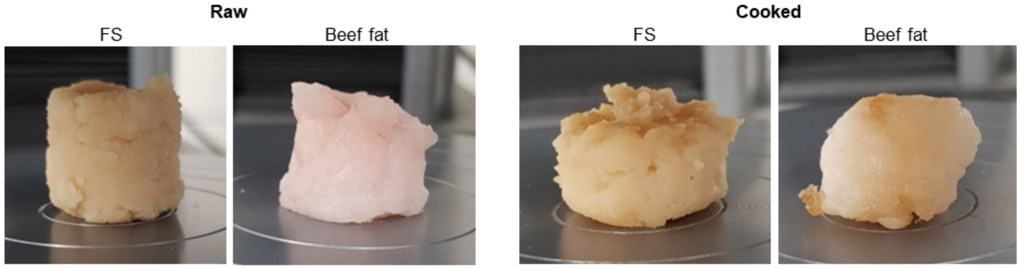

Supplementary Figure 4. Fat substitute. (a) TGA analysis of canola oil, GMS, and FS without protein presented as % weight against the temperature changes and (b) its 1st derivative. (c) DSC obtained onset temperature (To), peak temperature (Tp), and enthalpy (ΔH) of an FS and beef fat. (d) Textural and color properties of raw and cooked FS and beef fat. (e) The visual appearance of raw and cooked FS and beef fat. Source data are provided as a Source Data file.

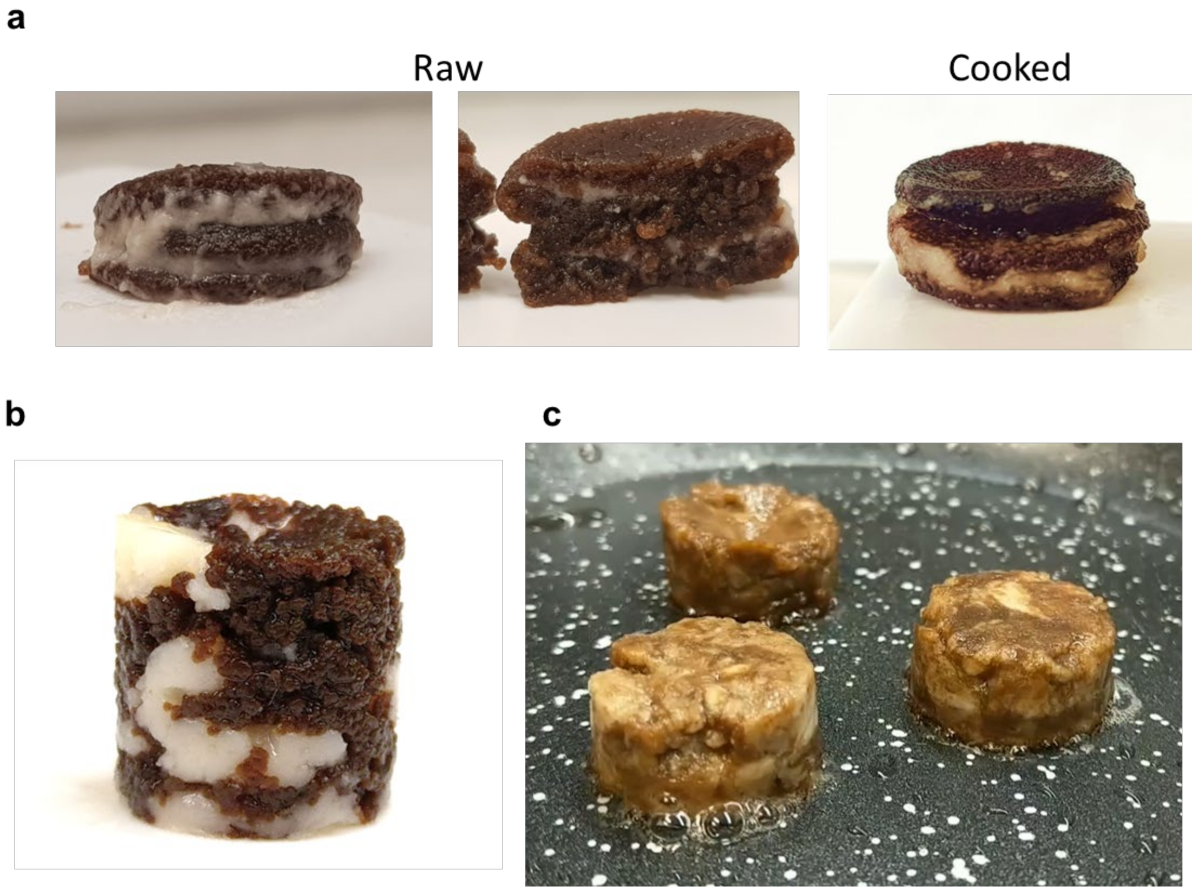

Supplementary Figure 5. Cultured meat prototype appearance. (a) The appearance of raw (whole and cut) and cooked layered CM prototypes. (b) Unorganized cellularized aggregates structured into a CM prototype. (c) Frying of the burger-like CM presented in Figure 5.

a

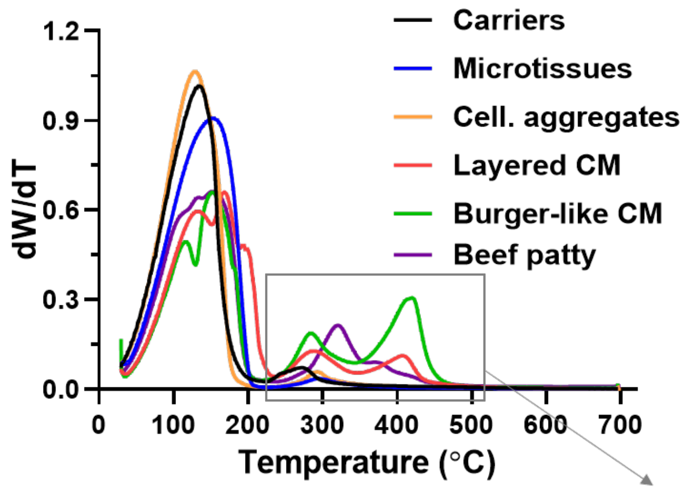

b

Samples	Stage	To (°C)	Tp (°C)	Te (°C)	Residue (%)
Carriers	I	254.9 ± 9.5	275.1 ± 4.8	295.3 ± 0.2	5.0 ± 1.4
Microtissues	I	235.2 ± 4.9	295.9 ± 1.9	352.0 ± 6.0	4.0 ± 0.6
Cell. aggregates	I	267.7 ± 1.9	295.2 ± 1.5	324.8 ± 6.8	3.9 ± 0.1
Layered CM	I	229.5 ± 6.4	284.1 ± 1.2	339.0 ± 8.5	
	II	349.0 ± 2.8	401.7 ± 3.6	439.0 ± 4.2	7.0 ± 0.8
Burger-like CM	I	253.0 ± 0.9	283.6 ± 1.6	340.2 ± 9.6	
	II	387.8 ± 1.0	411.1 ± 1.9	435.5 ± 3.4	4.9 ± 0.6
Beef patty	I	285.0 ± 0.4	316.8 ± 1.1	370.9 ± 12.8	6.3 ± 0.1

Supplementary Figure 6. (a) TGA 1st derivative and (b) TGA analysis of the microcarriers, microtissues, cellularized aggregates, layered CM, burger-like CM, and beef patty. Source data are provided as a Source Data file.

a

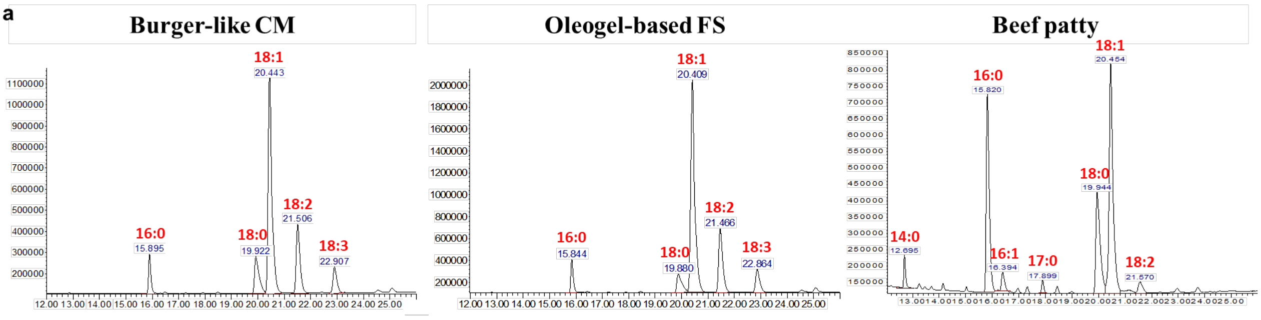

b

Fatty acid	14:0	16:0	16:1	17:0	18:0	18:1 w9	18:2 w6	18:3 w3
**Burger-like CM**	0	6.35	0	0	11.29	55.89	18.54	7.93
**Oleogel-based FS**	0	6.04	0	0	6.95	60.47	19.48	7.06
**Beef patty**	2.80	29.19	3.07	1.40	20.71	40.36	2.46	0

Supplementary Figure 7. Fatty acid profile of the CM prototype, oleogel-based FS, and beef patties. (a) GC-FID chromatograms and (b) fatty acid profile. Only the major peaks from (a) were considered for (b).

Supplementary Table 1. Timeline of the CM prototypes production.

	Cell seeding	Cell expansion	Aggregation	Structuring	Total
Burger-like CM prototype	24hr	8 days	-	7 days	16 days
Layered CM prototype (disc-like)	24hr	8 days	7 days	7 days	23 days
