# Supplementary material for: Cultured meat platform developed through the structuring of edible microcarrier-derived microtissues with oleogel-based fat substitute
Source: Nat Commun. 2023 May 23;14:2942. doi: 10.1038/s41467-023-38593-4 (PMC10205709; doi:10.1038/s41467-023-38593-4)
Supplement: Supplementary file 3 — Description of additional supplementary files [file 41467_2023_38593_MOESM3_ESM.pdf]

<u>Description of additional supplementary files</u>

Supplementary Movie 1 - Bioreactor foaming when aerated through gas sparging.

Supplementary Movie 2 - Gradually increasing the bioreactor stirring speed from 60 rpm to 80 rpm allows proper suspension of the cell microcarriers along culture.

Supplementary Movie 3 - Cooking of burger-like CM prototypes.
