# Supplementary material for: Cultured meat platform developed through the structuring of edible microcarrier-derived microtissues with oleogel-based fat substitute
Source: Nat Commun. 2023 May 23;14:2942. doi: 10.1038/s41467-023-38593-4 (PMC10205709; doi:10.1038/s41467-023-38593-4)
Supplement: Supplementary file 7 — Reporting Summary [file 41467_2023_38593_MOESM7_ESM.pdf]

nature portfolio

Corresponding author(s): Marcelle Machluf, Ayelet Fishman, Maya Davidovich-Pinhas

Last updated by author(s): 19.4.2023

Reporting Summary

Nature Portfolio wishes to improve the reproducibility of the work that we publish. This form provides structure for consistency and transparency in reporting. For further information on Nature Portfolio policies, see our Editorial Policies and the Editorial Policy Checklist.

Statistics

For all statistical analyses, confirm that the following items are present in the figure legend, table legend, main text, or Methods section.

n/a	Confirmed	
☐	☒	The exact sample size (*n*) for each experimental group/condition, given as a discrete number and unit of measurement
☐	☒	A statement on whether measurements were taken from distinct samples or whether the same sample was measured repeatedly
☐	☒	The statistical test(s) used AND whether they are one- or two-sided<br>*Only common tests should be described solely by name; describe more complex techniques in the Methods section.*
☒	☐	A description of all covariates tested
☒	☐	A description of any assumptions or corrections, such as tests of normality and adjustment for multiple comparisons
☐	☒	A full description of the statistical parameters including central tendency (e.g. means) or other basic estimates (e.g. regression coefficient) AND variation (e.g. standard deviation) or associated estimates of uncertainty (e.g. confidence intervals)
☐	☒	For null hypothesis testing, the test statistic (e.g. *F*, *t*, *r*) with confidence intervals, effect sizes, degrees of freedom and *P* value noted<br>*Give P values as exact values whenever suitable.*
☒	☐	For Bayesian analysis, information on the choice of priors and Markov chain Monte Carlo settings
☒	☐	For hierarchical and complex designs, identification of the appropriate level for tests and full reporting of outcomes
☒	☐	Estimates of effect sizes (e.g. Cohen's *d*, Pearson's *r*), indicating how they were calculated

Our web collection on statistics for biologists contains articles on many of the points above.

Software and code

Policy information about availability of computer code

Data collection	BD CellQuest Pro software (version 4.0.2, BD Biosciences, USA) and SkanIt software (for microplate readers, version 6.1.0.51, Thermo Scientific, USA) were used.
Data analysis	For the analyses, Excel (Microsoft Office Professional Plus 2019), ImageJ software (Fiji, version 1.53a, National Institute of Health, USA), FCS Express Flow Cytometry software (version 7.16.0035, De Novo Software, USA), NexyGen Lloyd software (version 4.1, Lloyd Instruments Ltd., UK), TA Universal Analysis software (version 5.1.0.46403, TA Instruments, USA), and Imaris software (version 9.0.2, Oxford Instruments, UK) were used. For the statistical analysis, GraphPad Prism 8 software (version 8.4.3, GraphPad Software, USA) was used. For the creation of schemes, BioRender.com was used.

For manuscripts utilizing custom algorithms or software that are central to the research but not yet described in published literature, software must be made available to editors and reviewers. We strongly encourage code deposition in a community repository (e.g. GitHub). See the Nature Portfolio guidelines for submitting code & software for further information.

Data

Policy information about availability of data

All manuscripts must include a data availability statement. This statement should provide the following information, where applicable:

- Accession codes, unique identifiers, or web links for publicly available datasets
- A description of any restrictions on data availability
- For clinical datasets or third party data, please ensure that the statement adheres to our policy

Source data are provided with this paper.

Human research participants

Policy information about studies involving human research participants and Sex and Gender in Research.

Reporting on sex and gender	N/A
Population characteristics	N/A
Recruitment	N/A
Ethics oversight	N/A

Note that full information on the approval of the study protocol must also be provided in the manuscript.

Field-specific reporting

Please select the one below that is the best fit for your research. If you are not sure, read the appropriate sections before making your selection.

☒ Life sciences ☐ Behavioural & social sciences ☐ Ecological, evolutionary & environmental sciences

For a reference copy of the document with all sections, see nature.com/documents/nr-reporting-summary-flat.pdf

Life sciences study design

All studies must disclose on these points even when the disclosure is negative.

Sample size	No sample-size calculation was performed. Sample size was determined for each analysis as the minimal sample size required to conclude meaningful conclusions such as differences or similarity between different groups, with a minimum of 3 samples per treatment.
Data exclusions	No data were excluded from the analyses.
Replication	The experiments were replicated or performed independently, according to the numbers mentioned in the manuscript (Methods section and figure legends).
Randomization	Samples, microcarriers, and cells were randomly allocated into experimental groups.
Blinding	Blinding is not relevant for the current work. The researchers were not blinded in the data acquisition. However, the data sets were objectively measured, hence blinding does not affect these data values.

Reporting for specific materials, systems and methods

We require information from authors about some types of materials, experimental systems and methods used in many studies. Here, indicate whether each material, system or method listed is relevant to your study. If you are not sure if a list item applies to your research, read the appropriate section before selecting a response.

Materials & experimental systems

n/a	Involved in the study
☐	☒ Antibodies
☒	☐ Eukaryotic cell lines
☒	☐ Palaeontology and archaeology
☒	☐ Animals and other organisms
☒	☐ Clinical data
☒	☐ Dual use research of concern

Methods

n/a	Involved in the study
☒	☐ ChIP-seq
☐	☒ Flow cytometry
☒	☐ MRI-based neuroimaging

Antibodies

Antibodies used	Conjugated primary antibodies for FACS and immunofluorescence staining: APC mouse anti-CD29 (Biolegend, 303008, TS2/16), APC rat anti-CD44 (Biolegend, 103011, IM7), PE mouse anti-CD45 (Invitrogen, MA1-81458, 1.11.32), APC Mouse IgG1, κ (BioLegend, 400120), APC Rat IgG2b, κ (BD Biosciences, 553991, A95-1), PE Mouse IgG1, κ (BioLegend, 400112, MOPC-21).
Validation	APC mouse anti-CD29 (https://www.biolegend.com/en-us/punchout/search-results/apc-anti-human-cd29-antibody-852?GroupID=BLG10310)<br>APC rat anti-CD44 (https://www.biolegend.com/fr-lu/products/apc-anti-mouse-human-cd44-antibody-312?GroupID=BLG10425)<br>PE mouse anti-CD45 (https://www.fishersci.com/shop/products/anti-cd45-rpe-clone-1-11-32/PIMA181458)<br>In all antibodies stainings, isotype control was used to rule out non-specific binding :<br>APC Mouse IgG1, κ (BioLegend, 400120, MOPC-21, https://www.biolegend.com/en-us/products/apc-mouse-igg1-kappa-isotype-ctrl-1404?GroupID=ImportedGROUP1).<br>APC Rat IgG2b, κ (BD Biosciences, 553991, A95-1, https://www.bdbiosciences.com/en-ca/products/reagents/flow-cytometry-reagents/research-reagents/flow-cytometry-controls-and-lysates/apc-rat-igg2b-isotype-control.553991)<br>PE Mouse IgG1, κ (BioLegend, 400112, MOPC-21, https://www.biolegend.com/en-us/products/pe-mouse-igg1-kappa-isotype-ctrl-1408)

Flow Cytometry

Plots

Confirm that:

☒ The axis labels state the marker and fluorochrome used (e.g. CD4-FITC).

☒ The axis scales are clearly visible. Include numbers along axes only for bottom left plot of group (a 'group' is an analysis of identical markers).

☒ All plots are contour plots with outliers or pseudocolor plots.

☒ A numerical value for number of cells or percentage (with statistics) is provided.

Methodology

Sample preparation	The bMSCs were trypsinized from the tissue culture plates at 80% confluency or from the microtissues. The detached cells were washed with the FACS buffer (5% chicken serum in PBS) twice by centrifugation for 6 min at 500 g. The cells were then resuspended in the FACS buffer and stained with APC mouse anti-CD29 (BioLegend, 303008, TS2/16, 1:200 dilution), APC rat anti-CD44 (BioLegend, 103011, IM7, 1:200 dilution), and PE mouse anti-CD45 (Invitrogen, MA1-81458, 1.11.32, 1:100 dilution) on ice for 45 min in the dark. The stained cells were finally washed twice and resuspended in the FACS buffer for flow cytometry.
Instrument	BD FACSCalibur™ Flow Cytometer (BD Biosciences, USA)
Software	BD CellQuest Pro software (BD Biosciences, USA) was used for data collection. FCS Express Flow Cytometry software (version 7.16.0035, De Novo Software, USA) was used for data analysis.
Cell population abundance	The population abundance of bovine mesenchymal stem cells were provided in Supplementary Fig. 1.
Gating strategy	The gating strategy is exemplified in Supplementary Fig. 1.

☒ Tick this box to confirm that a figure exemplifying the gating strategy is provided in the Supplementary Information.
